# Artificial intelligence based quantitative myocardial blood flow assessment facilitates the detection of multi-vessel coronary artery disease following vasodilator stress perfusion cardiovascular MR imaging

**DOI:** 10.1093/ehjimp/qyae045

**Published:** 2024-05-16

**Authors:** Georgios Moutzoukis, Benedikt Wagner, Marie K Lorenz, Heiko Mahrholdt, Andreas Seitz

**Affiliations:** Division of Cardiology and Angiology, Robert Bosch Medical Center, Auerbachstr. 110, 70376 Stuttgart, Germany; Division of Cardiology and Angiology, Robert Bosch Medical Center, Auerbachstr. 110, 70376 Stuttgart, Germany; Division of Cardiology and Angiology, Robert Bosch Medical Center, Auerbachstr. 110, 70376 Stuttgart, Germany; Division of Cardiology and Angiology, Robert Bosch Medical Center, Auerbachstr. 110, 70376 Stuttgart, Germany; Division of Cardiology and Angiology, Robert Bosch Medical Center, Auerbachstr. 110, 70376 Stuttgart, Germany

**Keywords:** myocardial blood flow, CTO, MBF, CMR, quantitative perfusion

A 67-year-old male presenting with atypical angina and exertional dyspnoea (NYHA II) underwent vasodilator stress perfusion cardiovascular magnetic resonance imaging (S-CMR) for work-up of clinically suspected coronary artery disease (CAD).

Baseline CMR revealed preserved left ventricular systolic function, as well as a focal area of subendocardial late gadolinium enhancement compatible with small inferior myocardial infarction. Following sufficient adenosine stress (HR increase by 20 bpm, SMBFmax > 1.43 mL/g/min, and MPR > 2 in at least one AHA-segment) standard visual analysis of first-pass perfusion images demonstrated an inferior wall defect extending beyond the infarcted area, yet still most likely representing single-vessel disease.

Subsequent invasive coronary angiography, however, uncovered severe triple-vessel disease with a collateralized chronic occlusion of the right coronary artery, as well as severe proximal stenosis of the proximal left anterior descending and the left circumflex arteries.

Interestingly, whereas standard visual perfusion analysis suggested single-vessel disease, new quantitative artificial intelligence based myocardial blood flow assessment—which we have performed additionally during initial CMR work-up for scientific reasons—demonstrated global impairment of hyperaemic myocardial blood flow (all 16 segments with SMBF < 1.94 mL/g/min) matching the angiographic findings of severe triple-vessel disease (*Figure 1*).

**Figure 1:**
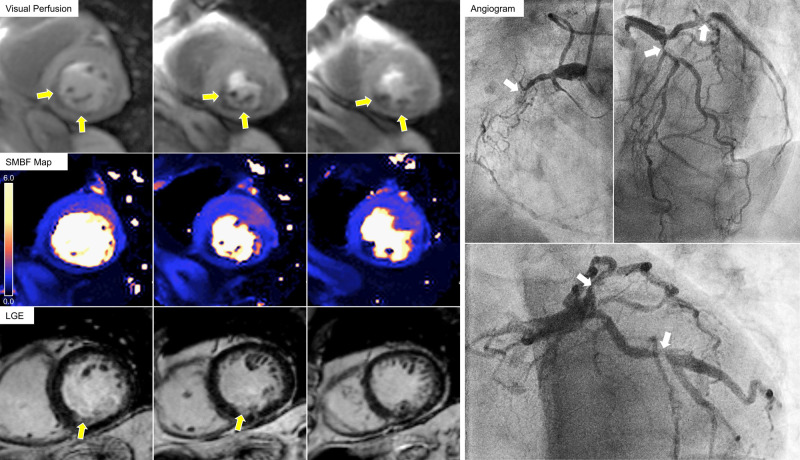
Left panels display CMR results: Visual stress perfusion CMR analysis demonstrating an inferior stress perfusion defect can be seen in the left top row (yellow arrows). LGE images are depicted in the left bottom row. Note that the focal inferior infarct (bottom row, yellow arrows) is much smaller than the stress perfusion defect visible in the top row, indicating inferior ischemia and thus most likely single vessel CAD. However, colour maps of the artificial intelligence based quantitative stress perfusion analysis (left middle row) reveal impaired myocardial blood flow in most segments (blue colour) despite sufficient adenosine stress, nicely matching invasive angiography results (right panels) demonstrating severe triple vessel CAD (white arrows).

Thus, our case underlines the potential risk of underestimation of the true extent of myocardium at risk and severity of CAD in patients with chronic coronary total occlusion plus additional stenosis using standard visual assessment of vasodilator S-CMR. The novel fully automated artificial intelligence based quantitative perfusion analysis may improve the sensitivity for detection of multi-vessel disease, thereby aiding clinical decision-making and achieving complete revascularization.

## Consent

Informed consent was given by the patient.

## Data Availability

The data underlying this article will be shared on reasonable request to the corresponding author.

## Lead author biography

Georgios Moutzoukis is a young cardiologist at Robert Bosch Hospital in Stuttgart, Germany. He has a particular interest in cardiovascular imaging and has specialized in Cardiac Magnetic Resonance (CMR). After completing his CMR-fellowship at Robert Bosch Hospital, he is now actively involved in research projects in the field of CMR.

